# Influence of Dairy Cows Bedding Material on the Microbial Structure and Antibiotic Resistance Genes of Milk

**DOI:** 10.3389/fmicb.2022.830333

**Published:** 2022-02-25

**Authors:** Haoming Wu, Yang Wang, Bingyao Du, Huiying Li, Lei Dong, Haiyan Hu, Lu Meng, Nan Zheng, Jiaqi Wang

**Affiliations:** ^1^State Key Laboratory of Animal Nutrition, Institute of Animal Sciences, Chinese Academy of Agricultural Sciences, Beijing, China; ^2^Laboratory of Quality and Safety Risk Assessment for Dairy Products of Ministry of Agriculture and Rural Affairs, Institute of Animal Sciences, Chinese Academy of Agricultural Sciences, Beijing, China; ^3^Key Laboratory of Quality and Safety Control for Milk and Dairy Products of Ministry of Agriculture and Rural Affairs, Institute of Animal Sciences, Chinese Academy of Agricultural Sciences, Beijing, China; ^4^State Key Laboratory of Membrane Biology, Tsinghua University-Peking University Joint Center for Life Sciences, School of Life Sciences, Tsinghua University, Beijing, China

**Keywords:** tank milk, bedding material, Illumina MiSeq, bacterial diversity, ARGs

## Abstract

The presence of pathogenic bacteria and antibiotic resistance genes (ARGs) in milk are among the most important issues related to the safety of dairy products and the health of consumers. However, despite that dairy cow are housed for long periods of time on different beddings, the effect of different bedding materials on the microbiota and presence of ARGs is unclear. In this study, the composition of microorganisms, and the presence of mastitis pathogens and 33 ARGs targeting seven antibiotics in raw milk produced from farms using sand bedding, rice husk bedding, and recycled manure solids (RMS) bedding were compared by amplicon sequencing and real-time quantitative PCR. The results showed that the microbial composition of milk was related to the microbiota of bedding. None of the mastitis pathogens were detected in milk from cows housed on sand bedding (S-M). The proportion of ARGs was highest in the S-M group and lowest in the milk from cows housed on RMS bedding (RMS-M) group. In general, the content of ARGs in RMS-M was the lowest, however, the RMS bedding may pose a threat to the breast health of dairy cows.

## Introduction

Milk is rich in nutrients, which not only supports human survival, but is also a good culture medium for microorganisms (Akindolire et al., 2018; Regasa et al., 2019). In addition to pathogenic bacteria, there may also be drug-resistant microorganisms in raw milk (Munsch-Alatossava and Alatossava, 2007; Caudell et al., 2018). Livestock feces can be used as an important potential source of pollution of pathogenic bacteria and antibiotic resistance genes (ARGs) in the environment. The use of antibiotics and epidemic prevention in intensive farming environments cause antibiotic pollution through feces, which further promotes the accumulation and spread of pathogenic bacteria and ARGs in the environment (Zhu et al., 2013; Wang et al., 2017). Multiple drug resistance genes were detected in manure samples, including tetracycline, sulfonamide, tylosin, macrolides, aminoglycosides, vancomycin, and macrolide lincomycin streptomycin B (Hölzel et al., 2010; Zhang et al., 2020).

Previous studies found that aerobic composting was a promising method to control pathogens and antibiotic contamination in feces (Du and Liu, 2012). However, although composting is an effective method to reduce the content of antibiotics and ARGs in animal feces, the risk of environmental exposure to ARGs in composted feces still exists (Selvam et al., 2012; Tien et al., 2017). In addition, sand and rice husk are often used as bedding materials for dairy cows. There is a small amount of microbiota in the sand bedding materials, and it has soft and comfortable performance, which can effectively maintain the breast health of dairy cows (Rowbotham and Ruegg, 2016b). And Rice husk bedding and other organic materials are easier to make pathogenic bacteria survive and grow (Hogan and Smith, 2012). However, the effect of bacteria in bedding on the bacteria and ARGs in raw milk remains unclear.

With increases in the number of dairy farms, there are an increasing number of large-scale pastures and dairy farming communities, and thus, the generation of fecal pollution has also increased greatly (Wang et al., 2018; Li et al., 2019). Fecal pollution not only occurs on the farm, where it provides a reservoir for mosquitoes and flies to breed and transmit disease, but also pollutes the air, soil, and water sources. Fecal pollution also restricts ranch production, pollutes the surrounding environment, endangers residents’ lives, and imparts great pressures on ranch managers.

Reasonable and effective treatment of cow manure and environmental protection has become an important topic in animal husbandry research. Since the 1970s, the United States and European Union countries have started the process of using recycled manure solids (RMS) as bedding material for dairy cows (Timms, 2008; Husfeldt et al., 2012). However, due to the existence of a large number of microorganisms in cow feces, the effect of RMS bedding on microorganisms in raw milk is unclear. The lack of knowledge surrounding the effect of RMS bedding on the microorganisms in raw milk restricts the popularization and application of RMS cushion-processing technologies. Therefore, it is urgent to study the impact of RMS on the microbiota and safety of raw milk compared to traditional bedding materials.

RMS cushions can be placed deep in the cow bed, like sand cushions, to increase the cow bedtime and reduce the occurrence of cow walking and joint injury (Tucker and Weary, 2004). RMS can effectively reduce the cost of pasture bedding and reduce the amount of pollution from cow dung in the environment. Manure-based bedding materials may cause harm to animals and human health, mainly due to the presence of a large number of microorganisms in the manure itself (Jahne et al., 2015). In previous studies, it was found that the processing of feces, such as extrusion, dehydration, fermentation, and drying, the microbial diversity in feces changed significantly, and the microbiota in the final material was significantly different from the original fecal microbiota (Wu et al., 2020). Most of the pathogenic bacteria in feces that cause mastitis are killed during the RMS processing and fermentation process, but some still remain (Wu et al., 2020). Some gram-negative bacteria, spore formers, heat-resistant bacteria, and food spoilage-related microorganisms, such as Pseudomonas, Bacillaceae, and Moraxellaceae, can remain in milk (Sorter et al., 2014; Leach et al., 2015; Rowbotham and Ruegg, 2016b; Wu et al., 2020). Although some studies have pointed out that the type of bedding will not affect the total bacterial count (TBC) in raw milk (Bradley et al., 2018). The effect of the bedding microbiota on milk microbiota is unclear.

The application of RMS bedding can reduce environmental pollution and the overall purchase cost of bedding; however, the impact of bedding on the safety of raw milk is not clear. The purpose of this study was to analyze the effects of different bedding materials on the microbial diversity of milk, as well as the pathogens and ARGs that raw milk contains.

## Materials and Methods

### Sample Collection

Bedding samples were collected from the same area as described in previous studies; from farms in Hebei, Heilongjiang, and Tianjin [Hebei: sand bedding (S-B); Heilongjiang: rice husk bedding (RH-B); Tianjin: RMS bedding (RMS-B) in healthy cowsheds] in October 2019 (Wu et al., 2020). The number of dairy cows in the three farms is about 5,000. Except for the types of bedding, the feed management and pasture facilities are similar. All pastures use milking parlor equipment to milk cows, and the milking process and method are the same. Bedding samples were collected from the center and four corners of the farms for 3 consecutive days, and five samples collected at the same time were mixed together. Raw milk samples were collected from the milk tanks in farms using rice husk beddings (RH-M), RMS bedding (RMS-M), and sand bedding (S-M) in the morning, noon, and evening after milking for 3 consecutive days. The three samples collected on the same day were mixed together. Samples were collected and stored directly on ice and transported to the laboratory. The samples were stored at −20°C until tested.

### DNA Extraction, Amplification, and Sequencing

DNA extraction was performed as described previously (Wu et al., 2020). In brief, a total of 200 mg of samples were mixed with 70% alcohol and PBS and centrifuged to remove impurities. The DNA was extracted using the E.Z.N.ATM Mag-Bind Soil DNA Kit (M5635-02, OMEGA). After DNA quantification using the qubit 2.0 DNA kit, PCR was performed using v3-v4 universal primers 341f (5'-cctacggcgwgcag-3') and 805r (5'-gatachvggggatctatcc-3'). DNA was amplified using a Bio-Rad T 100TM thermal cycler (California, United States). The amplified products were evaluated by 1.5% agar gel electrophoresis. The SanPrep DNA Gel Extraction Kit (SANGON Biotechnology, Shanghai, China) was used to extract and purify the amplified DNA. The Qubit2.0 DNA kit was used to accurately quantify the recovered DNA, and the final sequencing concentration was 20 pmol. The purified DNA products were sequenced on the Illumina MiSeq pe300 sequencing platform (California, United States) at SANGON Biotechnology Co., Ltd. (Shanghai, China).

### Bioinformatics

The forward and reverse sequences from the 16S rRNA amplicon gene were introduced into the qiime2-2021.04 system (Hall and Beiko, 2018). After confirming the data quality, dada2 plugin was used to remove the primer and chimeric sequences. After removing low-quality sequences, the sequence was spliced with each sequences. The spliced sequences were matched with 99% certainty to hits in the Greengene database to obtain bacterial taxonomic names. After adjusting all sample feature tables to 14,000 sequences, diversity analyses, including Shannon Diversity Index, Simpson Diversity Index, Chao1, and observed features were performed.

### qPCR Analysis of Pathogenic Bacteria and Antimicrobial Resistance Genes

In order to improve the sensitivity of qPCR and improve the quality of the DNA extraction, DNA extraction was performed by referring to previous methods (Mertens et al., 2014). After centrifugation of 1.5 ml of milk at 15,000 rpm for 10 min, the supernatant was removed. Cell degradation and DNA extraction were mediated by protease K and cetyltrimethylammonium bromide for 30 min at 60°C. The mixed solution was extracted and purified following to the manufacturer’s instructions for the DNeasy Blood and Tissue Kit (Qiagen, Germantown, MD, United States; Munsch-Alatossava and Alatossava, 2007). The amount and purity of the extracted DNA were determined by NanoDrop (Thermo Fisher, United States).

The detection of mastitis pathogens in dairy cows was performed as described in the Bovine Mastitis Pathogenic Bacteria Nucleic Acid Typing Detection Kit (Shenzhen Bioeasy Biotechnologies, Co., Ltd.; Wu et al., 2020). Real-time PCR based on TaqMan probes was used to detect the common contact infectious pathogens (*Staphylococcus aureus*, *Streptococcus agalactiae*, *Mycoplasma bovis*, *Corynebacterium bovis*, *Mycoplasma bovis*, *Mycobacterium bovis*, and *Mycoplasma* spp.), environmental pathogens (i.e., *Staphylococcus* spp., *Escherichia coli*, *Klebsiella* spp., *Prototheca* spp., *Streptococcus dysgalactiae*, *Streptococcus uberis*, *Trueperella pyogenes*, *Serratia marcescens*, and yeasts), and β-lactamase resistance genes in milk. Four fluorescence channels, fam, hex, Rox, and Cy5 were used to collect quantitative data.

qPCR experiments were performed for ARGs in milk samples using an ABI 7900 HT system. The final reaction volume was 20 μl, including 2 μl of DNA template and 10 μl of 2× RealStar Green Fast Mixture (GeneStar, Beijing, China). The amplification conditions were as follows: after denaturation at 95°C for 3 min, 40 cycles of 95°C for 15 s, 55°C for 30 s, and 72°C for 30 s were performed. The specific sequences of drug resistance genes were amplified and detected. Thirty-three antimicrobial resistance genes, including aminoglycosides (StrA and StrB), beta-lactamases (bla1, blaCMY, blaCTX-M-1, blaRoB, blaTEM, cfxA, and mecA), MLSB (ermA-1, ermA-2, ermA-3, ermB-1, ermB-2, ermC-1, and ermC-2), polymyxin (mcr-1), sulfonamides (Sul2), tetracyclines (tetA, tetB-1, tetB-2, tetC-1, tetC-2, tetH, tetQ, tetW-1, tetW-2, and tetW-3), and vancomycin (VanC-1, VanC-2, VanC-3, and VanG) were detected. The primers used to amplify the antimicrobial resistance genes were the same as those detailed previously (Table 1; Looft, 2012; Ouyang et al., 2015; Muurinen et al., 2021). The resistance genes were standardized to the 16S rRNA gene and quantified by amplification with 16S rRNA primers 357f (5'-cctacggaggcagg-3') and 517r (5'-attaccgcggctggg-3'; Selvam et al., 2012). After amplification, melt curves for all real-time PCR reactions were analyzed to determine the accuracy of the amplified target gene.

**Table 1:** qPCR primers for antibiotic resistance genes (ARGs).

Gene classification	Gene name	Forward primer (5'-3')	Reverse primer (5'-3')	References
*16srRNA*	357-518	CCTACGGGAGGCAGCAG	ATTACCGCGGCTGCTGG	Selvam et al., 2012
*Aminoglycosides*	StrA	CCGGTGGCATTTGAGAAAAA	GTGGCTCAACCTGCGAAAAG	Muurinen et al., 2021
*Aminoglycosides*	StrB	GCTCGGTCGTGAGAACAATCT	CAATTTCGGTCGCCTGGTAGT	Muurinen et al., 2021
*β-Lactamase*	bla1	GCAAGTTGAAGCGAAAGAAAAGA	TACCAGTATCAATCGCATATACACCTAA	Muurinen et al., 2021
*β-Lactamase*	blaCMY	CCGCGGCGAAATTAAGC	GCCACTGTTTGCCTGTCAGTT	Ouyang et al., 2015
*β-Lactamase*	blaCTX-M-1	GGAGGCGTGACGGCTTTT	TTCAGTGCGATCCAGACGAA	Ouyang et al., 2015
*β-Lactamase*	blaRoB	GCAAAGGCATGACGATTGC	CGCGCTGTTGTCGCTAAA	Ouyang et al., 2015
*β-Lactamase*	blaTEM	AGCATCTTACGGATGGCATGA	TCCTCCGATCGTTGTCAGAAGT	Ouyang et al., 2015
*β-Lactamase*	cfxA	TCATTCCTCGTTCAAGTTTTCAGA	TGCAGCACCAAGAGGAGATGT	Ouyang et al., 2015
*β-Lactamase*	mecA	GGTTACGGACAAGGTGAAATACTGAT	TGTCTTTTAATAAGTGAGGTGCGTTAATA	Ouyang et al., 2015
*MLSB*	ermA-1	CGGATCAGGAAAAGGACATTTT	AGCCTCCATCAATTTCTATAGCAGTAA	Looft, 2012
*MLSB*	ermA-2	CATTTTACCAAGGAACTTGTGGAA	TGGCATGACATAAACCTTCATCA	Looft, 2012
*MLSB*	ermA-3	AAATCGGATCAGGAAAAGGACAT	CCTCCATCAATTTCTATAGCAGTAACTG	Looft, 2012
*MLSB*	ermB-1	TGAAAGCCATGCGTCTGACA	CCCTAGTGTTCGGTGAATATCCA	Looft, 2012
*MLSB*	ermB-2	ATTCACCGAACACTAGGGTTGCT	CATTCCGCTGGCAGCTTAA	Looft, 2012
*MLSB*	ermC-1	CGTGGAATACGGGTTTGCTAA	TAGGATGAAAATATTCTCTTGGAACCAT	Looft, 2012
*MLSB*	ermC-2	ATATCTTTGAAATCGGCTCAGGAA	ATGGTCTATTTCAATGGCAGTTACG	Looft, 2012
*Polymyxin*	mcr-1	CGGTCAGTCCGTTTGTTC	CTTGGTCGGTCTGTA GGG	Liu et al., 2016
*Sulfonamides*	Sul2	TCATCTGCCAAACTCGTCGTTA	GTCAAAGAACGCCGCAATGT	Looft, 2012
*Tetracyclines*	tetA	CTCACCAGCCTGACCTCGAT	CACGTTGTTATAGAAGCCGCATAG	Looft, 2012
*Tetracyclines*	tetB-1	AGTGCGCTTTGGATGCTGTA	AGCCCCAGTAGCTCCTGTGA	Looft, 2012
*Tetracyclines*	tetB-2	GCCCAGTGCTGTTGTTGTCAT	TGAAAGCAAACGGCCTAAATACA	Looft, 2012
*Tetracyclines*	tetC-1	CATATCGCAATACATGCGAAAAA	AAAGCCGCGGTAAATAGCAA	Looft, 2012
*Tetracyclines*	tetC-2	ACTGGTAAGGTAAACGCCATTGTC	ATGCATAAACCAGCCATTGAGTAAG	Looft, 2012
*Tetracyclines*	tetH	TTTGGGTCATCTTACCAGCATTAA	TTGCGCATTATCATCGACAGA	Looft, 2012
*Tetracyclines*	tetQ	CGCCTCAGAAGTAAGTTCATACACTAAG	TCGTTCATGCGGATATTATCAGAAT	Looft, 2012
*Tetracyclines*	tetW-1	TCCTTCCAGTGGCACAGATGT	GCCCCATCTAAAACAGCCAAA	Looft, 2012
*Tetracyclines*	tetW-2	TTGCAGAACTAGGGAGCGTAGAT	AAAAGATGTCACTGCTGTCTGGATA	Looft, 2012
*Tetracyclines*	tetW-3	ATGAACATTCCCACCGTTATCTTT	ATATCGGCGGAGAGCTTATCC	Looft, 2012
*Vancomycin*	VanA	AAAAGGCTCTGAAAACGCAGTTAT	CGGCCGTTATCTTGTAAAAACAT	Looft, 2012
*Vancomycin*	VanC-1	ACAGGGATTGGCTATGAACCAT	TGACTGGCGATGATTTGACTATG	Looft, 2012
*Vancomycin*	VanC-2	CCTGCCACAATCGATCGTT	CGGCTTCATTCGGCTTGATA	Looft, 2012
*Vancomycin*	VanC-3	AAATCAATACTATGCCGGGCTTT	CCGACCGCTGCCATCA	Looft, 2012
*Vancomycin*	VanG	ATTTGAATTGGCAGGTATACAGGTTA	TGATTTGTCTTTGTCCATACATAATGC	Ouyang et al., 2015

### Statistical Analysis

Prism 8 was used to analyze the relative differences in ARGs between groups. One-way ANOVA was used to analyze the intergroup differences in the α-diversity index, ARGs, and bacterial communities. Canonical correspondence analysis (CCA) was performed using pass 4.02 software. Spearman correlation coefficients and heatmaps were performed between the bacterial communities and ARGs in milk, the microbiota in bedding and milk using Omicshare tools, a free online data analysis platform.^1^

## Results

### Milk Has Stable Bacterial α-Diversities

In the milk from cows housed on RMS bedding, rice husks, and sand bedding, an average of 19,233, 51,110, and 52,553 reads were obtained, respectively. Table 2 summarizes the results of the bacterial α-diversity indexes in bedding samples and milk samples. There was no significant difference in the Chao 1 index and observed features of milk bacteria under different bedding environments (*p* > 0.05). The Shannon index for microorganisms in the RMS-B group was significantly higher than that in RH-B and S-B (*p* < 0.05), while the Shannon index for milk bacteria was not different between the three bedding types (*p* > 0.05). In addition, the evenness index (Simpson index) of microbiota were no significant differences in the milk among the different bedding environments. These results shows that different bedding environments did not affect the diversity and evenness of the bacteria in milk.

**Table 2:** One-way ANOVA of bacterial α-diversities of the microbiota in milk and bedding from different farms.

	RMS		Rice husk		Sand		*p*	
	Milk	Bedding	Milk	Bedding	Milk	Bedding	Milk	Bedding
Chao 1	63 ± 33	247 ± 56	178 ± 167	298 ± 32	111 ± 45	212 ± 150	0.429	0.563
Observed features	63 ± 33	246 ± 56	135 ± 111	263 ± 25	92 ± 35	165 ± 94	0.488	0.223
Shannon	2.784 ± 0.314	6.584 ± 1.098	2.670 ± 1.049	4.440 ± 0.385	2.889 ± 0.403	3.365 ± 0.248	0.925	**0.003**
Simpson	0.695 ± 0.047	0.967 ± 0.038	0.681 ± 0.236	0.837 ± 0.047	0.750 ± 0.050	0.793 ± 0.047	0.824	**0.007**
Good’s coverage	1.000 ± 0.000	1.000 ± 0.000	0.997 ± 0.004	0.997 ± 0.000	0.998 ± 0.001	0.996 ± 0.004	0.305	0.179

All samples were analyzed at a depth of 14,000 reads. Data were described using mean ± SD.

### Microbiota Were Different in Milk From Cows Housed Under Different Bedding Conditions

The main bacterial phyla and families in milk samples and bedding samples were sorted and are summarized in Table 3 (family proportion >1%). There were significant differences in the microbiota of milk from farms using different bedding conditions. The proportion of *Firmicutes* in milk from the RMS-M group and S-M group was significantly higher than that of milk from the RH-M group (*p* = 0.001). Among those bacteria, *Streptococcaceae* and *Staphylococcaceae* were observed in a high proportion in the RMS-M and S-M groups (proportion >10%), and a high proportion of *Enterococcaceae* was found in the RMS-M group (proportion >10%). Conversely, the proportion of Proteobacteria in the RH-M group was the highest, and the proportion of *Enterobacteriaceae* was the highest (proportion >80%), being much higher than that of the RMS-M and S-M groups (*p* < 0.001). Although the value of *p* of One-way ANOVA analysis result was 0.052, the average proportion of *Moraxellaceae* in the RMS-M and S-M groups was as high as 35.0% and 34.2%, respectively, while the average proportion in the RH-M group was only 3.4%. The average content of *Pseudomonadaceae* in the S-M group was 16.0%, while that in the RH-M group was 4.9% and that in the RMS-M group was 1.0%.

**Table 3:** Relative read abundance of different bacterial community structures at the phylum and family levels in different groups.

	RMS (%)		Rich husk (%)		Sand (%)		*p*	
	Milk	Bedding	Milk	Bedding	Milk	Bedding	Milk	Bedding
**p__Firmicutes**	53.6 ± 13.1	3.5 ± 2.3	3.1 ± 2.9	13.2 ± 8.2	48.2 ± 10.6	15.6 ± 8.3	**0.001**	0.155
*f__Streptococcaceae*	20.9 ± 34.1	0.0 ± 0.0	2.1 ± 3.1	0.2 ± 0.3	35.8 ± 8.1	0.0 ± 0.0	0.207	0.336
*f__Enterococcaceae*	14.4 ± 24.9	0.0 ± 0.0	0.0 ± 0.0	0.0 ± 0.0	0.8 ± 0.1	0.1 ± 0.0	0.44	0.199
*f__Staphylococcaceae*	14.4 ± 21.9	0.1 ± 0.1	0.4 ± 0.2	2.8 ± 0.3	11.1 ± 2.9	2.3 ± 2.5	0.43	0.129
*f__Clostridiaceae*	1.2 ± 1.0	0.6 ± 0.7	0.0 ± 0.0	0.0 ± 0.0	0.0 ± 0.0	0.0 ± 0.0	0.056	0.165
*f__Leuconostocaceae*	0.7 ± 0.5	0.0 ± 0.0	0.0 ± 0.0	0.0 ± 0.0	0.0 ± 0.0	0.0 ± 0.0	0.061	ND
*f__Planococcaceae*	0.2 ± 0.2	1.7 ± 1.1	0.2 ± 0.1	1.4 ± 0.8	0.3 ± 0.3	7.9 ± 4.6	0.825	**0.048**
*f__Bacillaceae*	0.0 ± 0.0	0.0 ± 0.0	0.2 ± 0.0	8.3 ± 7.8	0.1 ± 0.1	4.9 ± 2.3	0.168	0.174
**p__Proteobacteria**	39.7 ± 14.6	51.6 ± 6.2	95.9 ± 2.8	72.6 ± 9.0	51.2 ± 11.0	74.5 ± 4.7	**0.002**	**0.011**
*f__Moraxellaceae*	35.0 ± 14	16.6 ± 18.5	3.5 ± 2.4	61.9 ± 9	34.2 ± 19.2	66.5 ± 7.6	0.052	**0.005**
*f__Enterobacteriaceae*	1.4 ± 1.3	0.0 ± 0.0	86.8 ± 6.5	2.6 ± 0.4	0.9 ± 0.2	6.6 ± 4.8	**<0.001**	0.073
*f__Brucellaceae*	1.0 ± 1.7	0.0 ± 0.0	0.0 ± 0.0	0.1 ± 0.0	0.0 ± 0.0	0.0 ± 0.0	0.397	**<0.001**
*f__Pseudomonadaceae*	1.0 ± 0.8	4.2 ± 1.1	4.9 ± 7.3	0.6 ± 0.1	16 ± 25.1	0.1 ± 0.0	0.491	**<0.001**
*f__Halomonadaceae*	0.5 ± 0.4	0.5 ± 0.8	0.1 ± 0.2	0.0 ± 0.0	0.0 ± 0.0	0.0 ± 0.0	0.151	0.356
*f__Hyphomicrobiaceae*	0.1 ± 0.1	1.9 ± 1.2	0.0 ± 0.0	0.0 ± 0.0	0.0 ± 0.0	0.0 ± 0.0	0.110	**0.024**
*f__Rhizobiaceae*	0.1 ± 0.1	0.9 ± 0.3	0.0 ± 0.0	0.0 ± 0.0	0.0 ± 0.0	0.0 ± 0.0	0.118	**<0.001**
*f__Xanthomonadaceae*	0.1 ± 0.1	7.1 ± 1.4	0.1 ± 0	1.6 ± 0.4	0.1 ± 0.1	0.0 ± 0.0	0.837	**<0.001**
*f__Phyllobacteriaceae*	0.0 ± 0.1	5.0 ± 3.1	0.0 ± 0.0	0.0 ± 0.0	0.0 ± 0.0	0.1 ± 0.0	0.422	**0.024**
*f__Comamonadaceae*	0.0 ± 0.0	0.2 ± 0.4	0.0 ± 0.0	3.4 ± 0.6	0.0 ± 0.0	0.8 ± 1.1	0.132	**0.005**
*f__Alteromonadaceae*	0.0 ± 0.0	3.3 ± 2.5	0.0 ± 0.0	0.1 ± 0.0	0.0 ± 0.0	0.0 ± 0.0	0.623	**0.048**
*f__Erythrobacteraceae*	0.0 ± 0.0	2.5 ± 0.5	0.0 ± 0.0	0.0 ± 0.0	0.0 ± 0.0	0.0 ± 0.0	ND	**<0.001**
*f__Alcaligenaceae*	0.0 ± 0.0	2.6 ± 1.8	0.0 ± 0.0	1.4 ± 1.4	0.0 ± 0.0	0.2 ± 0.4	ND	0.17
**p__Actinobacteria**	1.8 ± 2.3	5.7 ± 1.4	0.4 ± 0.7	8.3 ± 1.7	0.0 ± 0.0	9.3 ± 4.1	0.318	0.316
*f__Corynebacteriaceae*	1.2 ± 1.8	0.3 ± 0.3	0.0 ± 0.0	0.0 ± 0.0	0.0 ± 0.0	0.0 ± 0.0	0.349	0.064
*f__Micrococcaceae*	0.2 ± 0.4	0.2 ± 0.1	0.0 ± 0.0	7.4 ± 1.5	0.0 ± 0.0	9.1 ± 4.1	0.427	**0.011**
*f__Intrasporangiaceae*	0.1 ± 0.1	1.9 ± 0.8	0.0 ± 0.0	0.0 ± 0.0	0.0 ± 0.0	0.0 ± 0.0	0.245	**0.003**
**p__Bacteroidetes**	1.3 ± 1.4	28.5 ± 2.7	0.2 ± 0.2	5.8 ± 3.0	0.4 ± 0.3	0.4 ± 0.6	0.285	**<0.001**
*f__Flavobacteriaceae*	0.8 ± 1.1	11.0 ± 8.2	0.1 ± 0.1	2.4 ± 1.4	0.0 ± 0.0	0.2 ± 0.2	0.356	0.072
*f__Porphyromonadaceae*	0.2 ± 0.3	1.5 ± 1.9	0.0 ± 0.0	0.0 ± 0.0	0.0 ± 0.0	0.0 ± 0.0	0.286	0.21
*f__Weeksellaceae*	0.2 ± 0.1	1.9 ± 0.5	0.0 ± 0.0	0.4 ± 0.5	0.3 ± 0.3	0.0 ± 0.0	0.242	**0.003**
*f__Saprospiraceae*	0.1 ± 0.1	3.0 ± 1.3	0.0 ± 0.0	0.0 ± 0.0	0.0 ± 0.0	0.0 ± 0.0	0.239	**0.004**
*f__Marinilabiaceae*	0.1 ± 0.1	1.0 ± 1.4	0.0 ± 0.0	0.0 ± 0.0	0.0 ± 0.0	0.0 ± 0.0	0.422	0.309
*f__Cyclobacteriaceae*	0.0 ± 0.0	1.5 ± 0.7	0.0 ± 0.0	0.0 ± 0.0	0.0 ± 0.0	0.0 ± 0.0	0.422	**0.007**
*f__Flammeovirgaceae*	0.0 ± 0.0	1.1 ± 0.7	0.0 ± 0.0	0.0 ± 0.0	0.0 ± 0.0	0.0 ± 0.0	0.422	**0.024**
*f__Sphingobacteriaceae*	0.0 ± 0.0	3.6 ± 2.0	0.0 ± 0.0	3.0 ± 1.2	0.0 ± 0.0	0.2 ± 0.3	0.422	**0.044**
*f__Chitinophagaceae*	0.0 ± 0.0	1.1 ± 0.8	0.0 ± 0.0	0.0 ± 0.0	0.0 ± 0.0	0.0 ± 0.0	ND	**0.049**
**p__Cyanobacteria**	2.0 ± 1.5	0.1 ± 0.1	0.1 ± 0.1	0.0 ± 0.0	0.0 ± 0.0	0.0 ± 0.0	0.053	0.150
**p__Tenericutes**	1.2 ± 1.0	0.0 ± 0.0	0.0 ± 0.0	0.0 ± 0.0	0.0 ± 0.0	0.0 ± 0.0	0.079	ND
*f__Mycoplasmataceae*	1.2 ± 1.0	0.0 ± 0.0	0.0 ± 0.0	0.0 ± 0.0	0.0 ± 0.0	0.0 ± 0.0	0.079	ND
**p__Verrucomicrobia**	0.0 ± 0.0	0.7 ± 0.5	0.0 ± 0.0	0.0 ± 0.0	0.0 ± 0.0	0.0 ± 0.0	0.819	**0.041**
*f__Verrucomicrobiaceae*	0.0 ± 0.0	0.6 ± 0.5	0.0 ± 0.0	0.0 ± 0.0	0.0 ± 0.0	0.0 ± 0.0	0.722	0.076
**p__Thermi**	0.0 ± 0.0	0.9 ± 0.6	0.0 ± 0.0	0.0 ± 0.0	0.0 ± 0.0	0.0 ± 0.0	0.080	**0.024**
*f__Trueperaceae*	0.0 ± 0.0	0.9 ± 0.6	0.0 ± 0.0	0.0 ± 0.0	0.0 ± 0.0	0.0 ± 0.0	0.080	**0.024**
**p__Planctomycetes**	0.0 ± 0.0	2.4 ± 1.3	0.2 ± 0.2	0.0 ± 0.0	0.0 ± 0.0	0.0 ± 0.1	0.500	**0.011**
*f__Planctomycetaceae*	0.0 ± 0.0	0.8 ± 0.5	0.0 ± 0.0	0.0 ± 0.0	0.0 ± 0.0	0.0 ± 0.0	0.407	**0.032**
*f__Pirellulaceae*	0.0 ± 0.0	1.5 ± 0.7	0.1 ± 0.2	0.0 ± 0.0	0.0 ± 0.0	0.0 ± 0.0	0.516	**0.004**
**p__Chloroflexi**	0.0 ± 0.0	3.3 ± 2.5	0.0 ± 0.0	0.0 ± 0.0	0.0 ± 0.0	0.0 ± 0.1	0.195	0.055
*f__SHA*	0.0 ± 0.0	0.8 ± 0.6	0.0 ± 0.0	0.0 ± 0.0	0.0 ± 0.0	0.0 ± 0.0	ND	5.693
**p__Gemmatimonadetes**	0.0 ± 0.0	2.1 ± 0.6	0.0 ± 0.0	0.0 ± 0.0	0.0 ± 0.0	0.0 ± 0.0	0.111	**0.001**

Data were described using mean ± SD.

### Bedding Bacteria Will Not Contribute to the Main Microbiota in Milk

**Figure 1:**
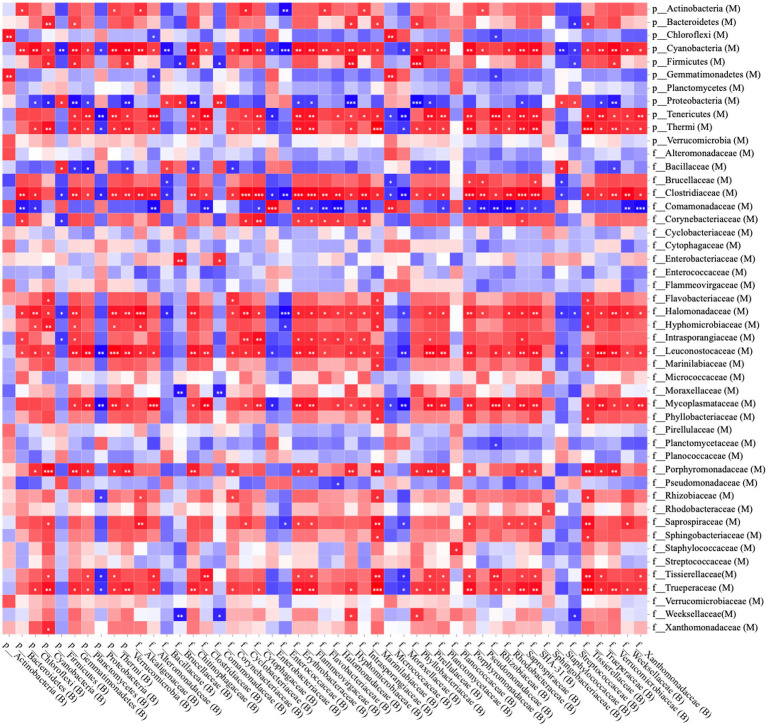
Spearman correlation analysis between bedding bacteria and milk bacteria at the phylum and family levels; red squares: positive correlation; blue squares: negative correlation; ^*^p < 0.05; ^**^p < 0.01; and ^***^p ≤ 0.001.

Following Spearman correlation analyses, the proportion of microorganisms in milk and bedding was summarized and transformed into a heatmap (Figure 1). The *Streptococcaceae*, *Staphylococcaceae*, *Enterococcaceae*, *Enterobacteriaceae*, and *Moraxellaceae* in milk samples were higher (proportion > 0.05%), but there was no significant correlation with the contents of the same bacterial family in bedding samples. *Cyanobacteria*, *Tenericutes*, and *Thermophiles*, were only found in the RMS-M and RMS-B groups (proportion > 0.05%), had a positive correlation with the same phyla in bedding samples (*p* < 0.05). *Clostridiaceae*, *Halomonadaceae*, *Intraporangiaceae*, *Porphyromonadaceae*, *Saprospiraceae*, *Marinilabiaceae*, and *Trueperaceae* were only found in the RMS-M and RMS-B groups (proportion > 0.05%), and the proportion of these family in bedding samples were positively correlated (*p* < 0.05). In addition, *Bacillaceae* were only detected in a large proportion of the RH-B (8.3% ± 7.8%) and S-B groups (4.9% ± 2.3%) and were detected in milk samples from the same farm. Thus, there was a positive correlation between the presence of *Bacillaceae* in milk samples and bedding samples (*p* < 0.05).

### Detection of Pathogenic Bacteria in Milk

The qPCR results of pathogenic microorganisms in milk are summarized in Table 4. Raw milk in milk tanks after milking in the morning, noon, and evening in the farms where different bedding materials were used for 3 consecutive days. The raw milk of the same day was mixed together. Regarding environmental pathogens, it was found that 1/3 of the RMS-M samples had strong positive results of *Enterococcus* spp. (+++), positive results of *Streptococcus dysgalatiae* (++), and weak positive results of yeast (+). A weak positive result (+) for *Escherichia coli* was detected in 1/3 of RH-M samples. qPCR of contact pathogens revealed that *Staphylococcus* spp. were found in all RMS-M samples, while *Mycoplasma bovis* was weakly positive (+) in 2/3 RMS-M samples. Samples that were positive (+) for *Mycoplasma* spp. and *Mycoplasma bovis* were detected in 1/3 of RH-M samples. A weak positive (+) result was detected for β-lactamase genes in all milk samples. No environmental pathogens or contact pathogens were detected in S-M samples.

**Table 4:** Detection of pathogenic bacteria in milk samples by qPCR.

Target bacteria gene	RMS			Rice husk			Sand		
	M1	M2	M3	M1	M2	M3	M1	M2	M3
Environmental pathogens									
*Enterococcus* spp. (Ensp)	**+++**	−	−	−	−	−	−	−	−
*Escherichia coli* (Ec)	−	−	−	−	**+**	−	−	−	−
*Klebsiella* spp. (klsp)	−	−	−	−	−	−	−	−	−
*Prototheca* spp. (Psp)	−	−	−	−	−	−	−	−	−
*Streptococcus uberis* (Sub)	−	−	−	−	−	−	−	−	−
*Serratia marcescens* (Sm)	−	−	−	−	−	−	−	−	−
Streptococcus dysgalactiae (Sdy)	**++**	−	−	−	−	−	−	−	−
*Trueperella pyogenes* (Tpy)	−	−	−	−	−	−	−	−	−
Yeast (Yea)	**+**	−	−	−	−	−	−	−	−
Contact pathogen									
*Mycoplasma* spp. (Mysp)	−	−	−	−	−	**++**	−	−	−
*Mycoplasma bovis* (Myb)	−	**+**	**+**	−	−	**++**	−	−	−
*Corynebacterium bovis* (Cb)	−	−	−	−	−	−	−	−	−
*Staphylococcus* spp. (Stsp)	**+**	**+**	**+**	−	−	−	−	−	−
*Staphylococcus aureus* (Sau)	−	−	−	−	−	−	−	−	−
*Streptococcus agalactiae* (Sag)	−	−	−	−	−	−	−	−	−
Others									
β-Lactamase resistance gene (Lac)	**+**	**+**	**+**	**+**	**+**	**+**	**+**	**+**	**+**

+++, strong positive; ++, positive; +, suspected positive; and −, negative.

### The Proportion of ARGs in the RMS-M Group Was the Lowest

**Figure 2:**
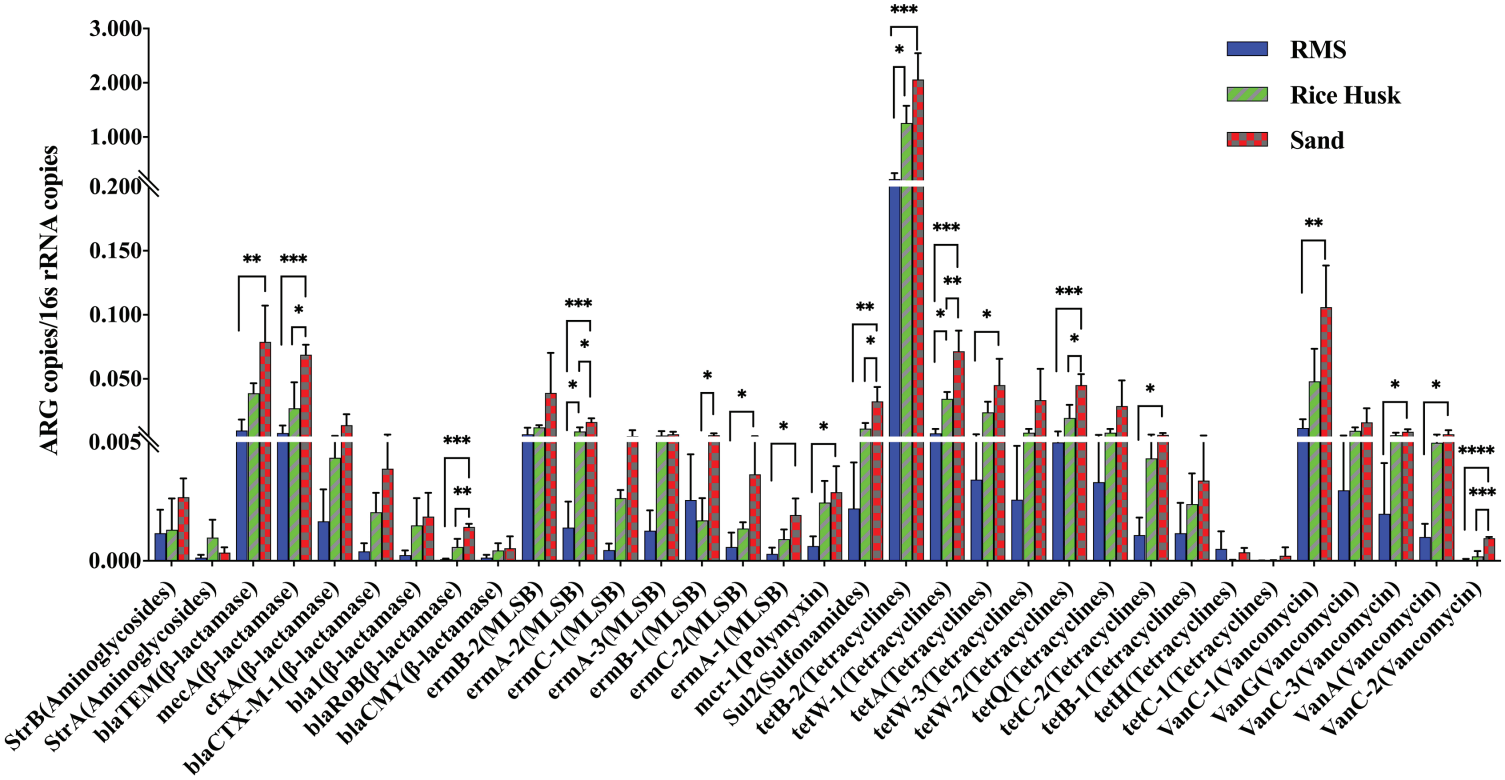
Detection proportion of ARGs detected in raw milk from different beddings. Data were described using mean ± SD. ^*^p < 0.05; ^**^p < 0.01; ^***^p ≤ 0.005; and ^****^p ≤ 0.001.

In this study, 33 ARGs targeting seven drugs were detected and analyzed (Figure 2). The proportion of ARGs targeting aminoglycosides in all milk samples was lower than .5%, with no significant differences between groups (*p* > 0.05). The content of the mcr-1 gene targeting polymyxin in milk samples was also lower than 0.005, however, its proportion in the S-M group was higher than that in the RMS-M group (*p* < 0.05). Similarly, the results revealed that in the S-M group, the proportion of ARGs genes targeting β-lactamases, MLSBs, sulfonamides, tetracyclines, and vancomycin was higher than that in RMS-M group (*p* < 0.05). There were also ARGs in the S-M group, including those targeting β-lactamases (mecA and blaRoB), MLSBs (emrA-2 and emrB-1), sulfonamides (sul2), tetracyclines (tetW-1 and tetW-2), and vancomycin (vanC-2), there were more of them in S-M than RH-M (*p* < 0.05). The proportion of MLSBs (ermB-2) and tetracyclines (tetB-2 and tetW-1) in the RH-M group was also significantly higher than that in the RMS-M group (*p* < 0.05).

### Relationship Between Milk Bacteria and ARGs

**Figure 3:**
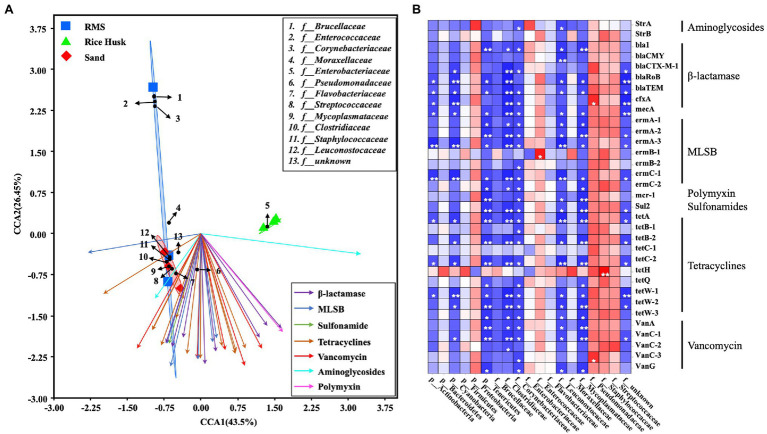
Combined analysis of ARGs and bacterial communities in milk. **(A)** Canonical correspondence analysis (CCA) of ARGs and bacterial family communities in milk. **(B)** Spearman correlation analysis between ARGs and milk bacteria at the phylum and family levels. Red squares: positive correlation; blue squares: negative correlation; ^*^p < 0.05; ^**^p < 0.01; and ^***^p ≤ 0.001.

The clustering relationship between the main microbiota of milk using different bedding environments was analyzed by CCA. At the same time, the relationship between the main microbiota and ARGs content in milk was analyzed by Spearman correlation analysis (Figure 3). The CCA results showed that the microbiota in the RH-M group was low similarity from that of the other two groups due to the high proportion of *Enterobacteriaceae*. From the heatmap of the correlation analysis, it was observed that *Enterococcaceae* in milk samples was positively correlated with ARGs (ermb-1) targeting MLSBs (*p* < 0.05). Additionally, the proportion of *Pseudomonadaceae* was positively correlated with ARGs (cfxa) targeting lactamases and vancomycin (vanc-3; *p* < 0.05), and the presence of *Staphylococcaceae* was positively correlated with ARGs targeting tetracyclines (*p* < 0.01). *Cyanobacteria* and *Tenericutes* had a significant negative correlation with multiple ARGs. *Cyanobacteria* and *Tenericutes* were only detected in RMS bedding (proportion >1%), and the proportion of these phyla in bedding was positively correlated with that in milk (Figure 3).

## Discussion

In this study, the contents of microbiota, pathogenic microorganisms, and ARGs in milk and RMS, rice husk, and sand bedding were collected and compared. As the surface where cow breasts contact for substantial periods of time, bedding materials and microorganisms in bedding, have a far-reaching impact on cow health and milk quality (Robles et al., 2020). Previous studies have confirmed that increases in the TBC in bedding are related to the TBC in the dairy cows udder skin (Rowbotham and Ruegg, 2016b). Microorganisms in beddings can pollute raw milk, thus, affecting its quality (Murphy et al., 2019). Some studies also pointed out that there was no significant difference in TBC content in milk under different bedding environments (Bradley et al., 2018). However, the effect of bedding microbiota on the milk microbiota is unclear.

In this study, it was found that there were a large number of *Enterobacteriaceae* (proportion >1%) in milk (RH-M) and bedding (RH-B) from farms using rice husk bedding. The use of sawdust bedding may be related to the animal epidemiology of coliform mastitis (Carroll and Jasper, 1980). In addition, a large number of *Streptococcaceae*, *Staphylococcaceae*, and *Enterococcaceae* were found in RMS-M group samples (proportion >10%). Previous studies also found that RMS bedding may increase the prevalence of *Streptococcus thermophilus* and *Enterococcus* in raw milk (Rowbotham and Ruegg, 2016a; Gagnon et al., 2020). Based on correlation analyses between bedding and milk microbiota, the presence of *Bacillaceae* in milk may be from the bedding (Figure 1). Those findings were also consistent with those found in previous studies (Driehuis et al., 2013; Skeie et al., 2019). It should be noted that the proportion of *Moraxellaceae* in all bedding and milk samples was greater than 1%. Previous studies also found that environmental *Moraxellaceae* may contaminate milk. *Moraxellaceae* can reproduce at low temperatures, secreting proteases, and lipases to alter the taste and odor of milk protein, thus, shortening the shelf life of milk and reducing its quality (Wu et al., 2019).

Sand cushion is considered to be the most ideal bedding material (Rowbotham and Ruegg, 2016a). The results of this study also revealed that no pathogenic bacteria were detected in the milk (S-M) produced from cows housed on sand bedding. Conversely, some environmental pathogens and contact pathogens were detected in RMS-M group, however.

Raw milk that has not been sterilized is not allowed to be sold in many countries. After the sterilization of raw milk in factories, pathogenic bacteria are killed. Of course, this does not mean that TBC in raw milk has no effect on the product. Compared with the threat of pathogenic bacteria to consumers, the drug resistance genes remaining in milk after processing may be more threatening. The prevalence of antibiotic resistance (AMR) continues to pose a global threat to human health (Ferroni et al., 2020). A recent study found that the direct transmission of microorganisms in raw milk is the main factor influencing the prevalence of AMR (Caudell et al., 2018), and raw milk plays an important role in maintaining the repository and spread of ARGs (Liu et al., 2020). In this study, S-M contained a higher proportion of ARGs than RMS-M (*p* < 0.05). The ARGs detected in the S-M group, including those targeting β-lactamases, MLSBs, sulfonamides, tetracyclines, and vancomycin, were significantly higher than those in RMS-M group samples. Some high level of ARGs may be caused by the antibiotic drugs used on farms, but it is unlikely that so many antibiotic drugs are used at the same time.

Low moisture content in sand bedding material can reduce the diversity and concentration of microbial species. Previous studies found that microorganisms evolve faster under harsh living environment conditions (Li et al., 2014). In addition, studies have also shown that extreme living environment can increase microbial drug resistance (Patel and Amaresan, 2014). Therefore, we hypothesize that the use of sand bedding may increase the prevalence of drug-resistant bacteria and affect the prevalence of drug resistance genes in raw milk.

Correlation analyses between the microbial composition of milk and the presence of ARGs found that *Pseudomonadaceae* had a significant positive correlation with some ARGs targeting β-lactamases and vancomycin, while a positive correlation was also found between *Enterococcaceae* and MLSBs, and *Staphylococcaceae* and tetracyclines. No positive correlations were found between other families and ARGs. In other studies, it was also found that *Pseudomonadaceae*, *Enterococcaceae*, and *Staphylococcaceae* have significant drug resistance and may have adverse effects on consumers (Morandi et al., 2006; Quintieri et al., 2019; Machado et al., 2020). In this study, we found that the changes of these bacteria in milk may be related to the bedding material. Therefore, monitoring the content of individual microorganisms alone may not be sufficient to monitor ARGs in raw milk. This study also had some deficiencies. The joint analysis of influencing milk bacterial factors, such as bedding type with season and service time, need to be further studied.

## Conclusion

In conclusion, this study compared the microbial composition of milk produced by cows housed on RMS bedding, sand bedding, and rice husk bedding. Microbial composition in cow milk under different bedding environments was significant differences. Pathogenic bacteria known to be involved in mastitis were found in the milk from cows housed on RMS bedding and rice husk bedding, but the ARGs in milk from cows housed on RMS bedding were significantly lower than those in milk from cows housed on sand bedding. These findings suggest that although RMS bedding may lead to the contamination of pathogenic microorganisms in milk, they may play a positive role in reducing the presence of ARGs in milk and, thus, protect the health of consumers.

## Data Availability Statement

The original contributions presented in the study are included in the article/supplementary material, further inquiries can be directed to the corresponding authors.

## Author Contributions

HW and YW: conceptualization, methodology, investigation, and manuscript writing, review, and editing. HL and BD: formal analysis. LD, HH, and LM: resources. NZ: data curation. JW: supervision and project administration. JW and HW: funding acquisition. All authors have read and agreed to the published version of the manuscript.

## Funding

This research was funded by the China Postdoctoral Science Foundation (grant number 2020TQ0357), the Scientific Research Project for Major Achievements of Agricultural Science and Technology Innovation Program (grant number CAAS-ZDXT2019004), the Agricultural Science and Technology Innovation Program (grant number ASTIP-IAS12), and the China Agriculture Research System of MOF and MARA.

## Conflict of Interest

The authors declare that the research was conducted in the absence of any commercial or financial relationships that could be construed as a potential conflict of interest.

## Publisher’s Note

All claims expressed in this article are solely those of the authors and do not necessarily represent those of their affiliated organizations, or those of the publisher, the editors and the reviewers. Any product that may be evaluated in this article, or claim that may be made by its manufacturer, is not guaranteed or endorsed by the publisher.

## Footnotes

^1^ https://www.omicshare.com/tools
